# Mapacalcine Protects Mouse Neurons against Hypoxia by Blocking Cell Calcium Overload

**DOI:** 10.1371/journal.pone.0066194

**Published:** 2013-07-02

**Authors:** Hamid Moha ou Maati, Catherine Widmann, Djamila Sedjelmaci Bernard Gallois, Catherine Heurteaux, Marc Borsotto, Michel Hugues

**Affiliations:** 1 Institut de Pharmacologie Moléculaire et Cellulaire, Centre National de la Recherche Scientifique (UMR7275), Université de Nice Sophia Antipolis, Valbonne, France; 2 Chimie Biologie des Membranes et des Nanoobjets, Centre National de la Recherche Scientifique (UMR5248), Pessac, France; Aston University, United Kingdom

## Abstract

Stroke is one of a major cause of death and adult disability. Despite intense researches, treatment for stroke remains reduced to fibrinolysis, a technique useful for less than 10% of patients. Finding molecules able to treat or at least to decrease the deleterious consequences of stroke is an urgent need. Here, we showed that mapacalcine, a homodimeric peptide purified from the marine sponge *Cliona vastifica*, is able to protect mouse cortical neurons against hypoxia. We have also identified a subtype of L-type calcium channel as a target for mapacalcine and we showed that the channel has to be open for mapacalcine binding. The two main L-type subunits at the brain level are CaV1.3 and CaV1.2 subunits but mapacalcine was unable to block these calcium channels.Mapacalcine did not interfere with N-, P/Q- and R-type calcium channels. The protective effect was studied by measuring internal calcium level variation triggered by Oxygen Glucose Deprivation protocol, which mimics stroke, or glutamate stimulation. We showed that NMDA/AMPA receptors are not involved in the mapacalcine protection. The protective effect was confirmed by measuring the cell survival rate after Oxygen Glucose Deprivation condition. Our data indicate that mapacalcine is a promising molecule for stroke treatment.

## Introduction

Despite decades of research, the prognosis for patients with brain ischemia remains poor. With an incidence of approximately 250–400 in 100000 and a mortality rate of around 30%, stroke remains the third leading cause of death, and a major cause of long-term disability and depression in industrialized countries [1]. During ischemic stroke, brain cells suffer from oxygen and glucose deprivation (OGD). This results in a dramatic neuronal depolarization, release of excitatory neurotransmitters, and reduced neurotransmitter re-uptake from the synaptic space. This process leads, *in fine,* to an excessive intracellular accumulation of Ca^2+^. Although cells can use different mechanisms to lower their internal Ca^2+^, they are unable to maintain Ca^2+^ homeostasis under ischemic stroke. Once internal Ca^2+^ is no more regulated, a variety of biological events occurs which leads to cell death. NMDA receptors, the most important excitatory neurotransmitter receptors in the central nervous system [2], [3], have long been considered as the main target responsible for Ca^2+^ overload in the ischemic brain [4], [5], [6], [7]. NMDA receptors interact with a wide variety of cellular proteins [8], [9], [10]. Thus they are linked to distinct downstream signaling molecules, including pathways involved in neurotoxicity. When brain cells die, function of the body parts they control is impaired or lost, causing paralysis, speech and sensory problems, memory and reasoning deficits, coma, and possibly death. Besides the dramatic clinical aspects of the disease, stroke and subsequent neurological injuries together represent a considerable financial burden in medical and rehabilitation expenses and a loss of productivity. Several therapeutics efforts using NMDA receptor or calcium antagonists have been disappointing [11], [12], [13], [14], [15]. Despite substantial research in neuroprotection, dozens of clinical trials have failed to show efficacy in humans for a variety of neuroprotective drugs and, to date, no efficient agent has been conclusively shown to be clinically effective in acute stroke. Currently, there are no effective, clinically approved methods that promote restoration of central nervous system (CNS) function, days, weeks or months after stroke. The discovery of new therapeutic strategies therefore represents an important challenge. A small homodimeric protein, the mapacalcine, (M.W. 19 KDa; P86916) produced by a marine sponge (*Cliona vastifica*) has been described as a specific blocker of calcium influxes, particularly resistant to all known calcium channel blockers [16]. Specific receptors for this protein have been detected in various tissues: intestinal smooth muscle, brain, kidney, and liver [16]–[20]. Among these studies, those conducted on cultured hepatocytes, demonstrated that mapacalcine was able to completely inhibit a calcium influx triggered by ischemia/reperfusion without affecting cell viability [17]. Mapacalcine and its cellular target therefore represent an interesting pathway for drug discovery in the domain of neuronal protection during stroke. Moreover the mapacalcine and its receptor may represent a starting point towards the understanding of one mechanism leading to cell death after stroke. In this paper using different approaches, we demonstrated that i) mapacalcine is able to inhibit cell calcium influx in mouse cortical embryonic neurons under different conditions occurring during stroke, ii) the target of mapacalcine is, at least in part, a subtype of L-type calcium channel and mapacalcine binds only to channels in an opened state iii) neuron survival after OGD is enhanced when cells are treated with mapacalcine before or after OGD. These data are in good agreement with previous report suggesting for mapacalcine a protective role against post ischemic cell calcium invasion [17].

## Materials and Methods

### Animals

All experiments were performed according to policies on the care and use of laboratory animals of European Community laws. The local Ethics Committee CIEPAL Azur (Comité Institutionnel d'Ethique Pour l'Animal de Laboratoire Côte d'Azur) approved the experiments (protocol numbers NCA/2006/10-1, NCA/2006/10-2 and NCE2008-08/09-0). All efforts were made to minimize animal suffering and reduce the number of animals used. Mice were housed under controlled laboratory conditions with a 12-hour dark-light cycle, a temperature of 21±2°C, and a humidity of 60 to 70%. Mice had free access to standard rodent diet and tap water.

### Primary culture of cortical embryonic mouse neurons

Time-pregnant (E14) C57Bl6/J mice were anesthetized with isopentane followed by cervical dislocation as previously described [21]. Fetuses were removed and placed in cold HBSS^+^ solution. Cerebral cortices were dissected in cold HBSS^+^ solution and the meninges removed. Cortical samples were cut in small pieces and were gently triturated with a fire-polished glass Pasteur pipette in 8 ml HBSS^+^ solution. The mix was filtered (40 µm filter) and centrifuged at 800 rpm for 8 min. The supernatant was removed and the pellet resuspended in 2 ml culture medium. Cells were plated on poly-D-lysine (Sigma-Aldrich Chimie, St Quentin Fallavier, France)-coated 35 mm diameter plates (CML, Nemours, France) at a density of 1×10^6^ cells/well in Neurobasal medium supplemented with B27, Glutamax, 100 units/ml penicillin, and 100 µg/ml streptomycin. Cultures were maintained at 37°C in a humidified incubator containing 5% CO_2_ and 95% air. Glial growth was suppressed by addition of 5-Fluoro-2-deoxyuridine (2 µM) during the second day of culture. Cultures were used for experiments between 10 and 12 days.

### Electrophysiology

#### Whole cell current recordings

All electrophysiological experiments were conducted on cortical embryonic neurons seeded at a density of 1,000,000 cells/35-mm dish after 10–12 days of culture. Recordings were performed in whole cell configuration of the patch clamp technique [22]. Each current was evaluated by using a RK 400 patch clamp amplifier (Axon Instrument, USA), low-pass filtered at 3 kHz and digitized at 10 kHz using a 12-bit analog-to-digital converter digidata (1322 series, Axon Instrument, USA). All current amplitudes are expressed in current densities. Results are expressed as mean ± standard error of the mean (SEM). Patch clamp pipettes were pulled using vertical puller (PC-10, Narishige) from borosilicate glass capillaries and had a resistance of 3–5 MΩ. The bath solution contained (in mM) 140 TEA-Cl, 10 BaCl_2_, 1 MgCl_2_, 4 4-AP, 10 HEPES, 10 Glucose, 10^−7^ TTX (pH 7.35 with TEA-OH). The pipette solution contained (in mM) 110 CsCl, 20 TEA-Cl, 3 Na_2_-ATP, 3.5 MgCl_2_, 10 EGTA, 10 HEPES (pH 7.25 with Cs-OH). All experiments were performed at room temperature (21–22°C). Stimulation protocols and data acquisition were carried out using a microcomputer (Dell Pentium) with a commercial software and hardware (pClamp 8.2). Calcium currents were recorded by voltage clamp steps to membrane potentials of –70 to +50 mV in 10 mV steps applied from a holding potential of −80 mV. Duration of depolarization pulses were 500 ms, and the pulse cycling rate was 2 s. Current amplitudes were evaluated at different potentials (−30, −20, −10, 0, 10 and 20 mV). In all electrophysiological experiments, we used the different drugs at the following concentrations: mapacalcine, 1 µM, nifedipine, 1 µM, SNX485, 250 nM, ω-conotoxin GVIA, 1 µM, and calcicludine, 75 nM. n = 10 per group.

#### Channel state dependent inhibition

In order to check whether the mapacalcine calcium channel inhibition is dependent on the opened or closed state of the channel, electrophysiological experiments were performed in the following conditions. Channels were stimulated with single repeated pulse from −80 mV holding potential to −10 mV during 0.5 s with a frequency rate of 5 s. Currents were recorded with two stimulation protocols. During the first protocol (permanent stimulation), channels were continuously stimulated and 1 µM mapacalcine solution was superfused at the 13^th^ stimulation and following. During the second protocol (delayed protocol), channels were stimulated for 12 cycles, and then mapacalcine solution was superfused for 50 seconds (corresponding to ten putative stimulation cycles) without stimulation. After this delay channels were stimulated for 15 new cycles. Data are expressed in current densities percentage ± SEM. The number of tested cells was 10(n = 10) per group.

#### Whole cell NMDA/AMPA recordings on mouse cortical neurons

NMDA/AMPA recordings were performed on neurons after 12–15 days of culture. Cells were seeded at a density of 1,000,000 cells/35 mm dish and cultured at 37°C in humidified incubator in an atmosphere of 95% air/5% CO_2_. NMDA/AMPA current recordings were realized using the gap free mode in the whole cell configuration of the patch clamp technique. In brief, after the whole configuration obtained, neurons were continuously clamped at −80 mV and the different drugs were tested. Extracellular solution contained (in mM): NaCl 150, KCl 5, HEPES 10, CaCl_2_ 0.2, glucose 10 and sucrose 10(pH 7.4 with NaOH). Pipette solution contained (in mM): CsCl 140, EGTA 10, HEPES 10, and Mg_2_-ATP 4 (pH 7.22 with CsOH). Cells were perfused with control solution during two minutes and then with the glutamate (100 µM) solution alone or additioned with 1 µM of Mapacalcine. Similar groups were tested by replacing glutamate by NMDA 100 µM. The nature of the glutamate current was checked using specific inhibitors of NMDA/AMPA receptors [respectively APV 50 µM ((2R)-amino-5-phosphonovaleric acid) and CNQX 10 µM (6-cyano-7-nitroquinoxaline-2,3-dione)]. Effects of Mapacalcine perfused alone or in the presence of 100 µM of glutamate were also measured. The glutamate currents on cortical neurons at 4–8 days of culture were also checked. The number of tested cells was 6 (n = 6) per condition.

#### Whole cell Cav 1.2/Cav 1.3 recordings on transfected HEK-293 cells

The different subunits of the human calcium channels (CACNA 1C/Cav 1.2 (α1); CACNA 1D/Cav 1.3 (α1); CACNA 2D1/α2δ1 and CACN1B/β1B) were subcloned into the pCDNA6 vector. On day one, HEK-293 cells were seeded at a density of 20000 cells per 35 mm dish. On day two, cells were transfected (1.5 µg of α1 Cav 1.2 or Cav 1.3+1 µg of α2δ1 +1 µg of β1B and 0.1 µg of pIRES2-GFP) using the JetPEI (Polyplus Transfection, France) technique according to the protocol given by the manufacturer. On day three, cells were dissociated and 10 new 35 mm dishes were prepared from one original 35 mm dish. On day four, cells were placed at 30°C for at least 24 hours. From this stage calcium currents can be recorded up to 6 days. The patch clamp extracellular and pipette media and the stimulation protocol for the Cav 1.2 and Cav 1.3 current recordings were the same as those used for neuron calcium current recording conditions. Calcium currents were recorded in control condition or in the presence of 1 µM of mapacalcine (n = 4 per condition). Effect of Mapacalcine on calcium current was evaluated at −10 mV.

### Oxygen Glucose Deprivation (OGD) model

OGD experiments were performed on primary mouse cortical neurons seeded at a density of 1,000,000 cells/35-mm dish between 10–12 days of culture [23]. The culture medium was replaced by thorough exchange with deoxygenated glucose-free Earl's balanced salt solution (BSS). Composition of BSS solution was (in mM): 140 NaCl, 5.4 KCl, 1.2 CaCl_2_, 0.9 MgCl_2_, 0.44 KH_2_PO_4_, 4.17 NaHCO_3_ and 0.34 Na_2_HPO_4_. Prior to use, BSS was equilibrated with the anaerobic gas mixture (5% CO_2_, 1.2% O_2_ and 93.8% N_2_) by bubbling for 15 min, adjusted to pH 7.4 if necessary, and heated to 37°C as described in [24]. Oxygen content of BSS was monitored with two oxygen-sensitive Clark electrodes and the Labchart 7.02 software (Powerlab, Oxford, UK). Cortical cells were subjected to 120 min OGD. 4 different cultures (n = 4) were tested.

### Cell calcium imaging

**Figure 1:**
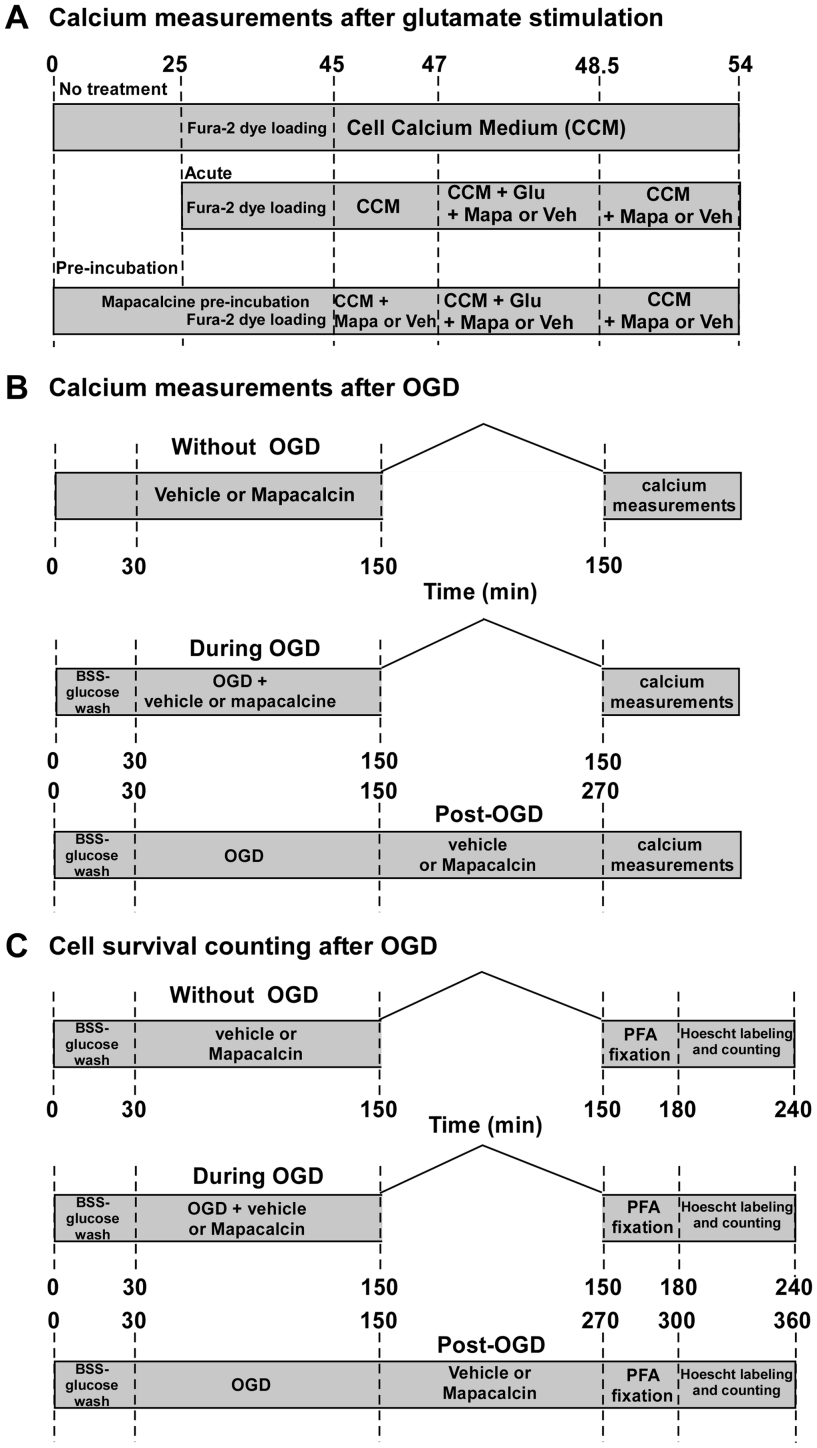
Glutamate and Oxygen Glucose Deprivation (**OGD**) **protocols.** Schematic representation of protocols used for internal calcium measures following 100 µM glutamate stimulation (A) or following OGD experiments (B). Cell survival counting protocols are also depicted (C). In these experiments, the vehicle was water. CCM, Cell Calcium Medium, Mapa, mapacalcine, PFA, 4% ParaFormAldehyde.

Mapacalcine effect was evaluated on intracellular calcium level. Calcium entry was triggered either by 100 µM glutamate stimulation or applying OGD protocol. All the different protocols used are summarized in Figure 1.

In the first protocol (glutamate), we performed 3 types of experiment (Figure 1A): i) *no treatment*, cells loaded with the Fura-2 dye and no further treatment was applied. As previously described [24] calcium levels were measured after the 20 min of dye loading followed by 2 min wash in the cell calcium medium (recovery period) which also contained mapacalcine in the experiments of pre-incubation. These measures corresponded to basal level of intracellular calcium. ii) *acute mapacalcine*, mapacalcine was applied at the same time as 100 µM glutamate. iii) *pre-incubation of mapacalcine*, in this protocol mapacalcine was applied 45 min before starting calcium measurements, this time allowing the equilibrium binding of mapacalcine to its receptor [20]. The dye loading period corresponded to last 20 min of pre-incubation. 9 different dishes (n = 9) were measured for each experimental condition.

In the second protocol (OGD), we also used 3 types of experiment (Figure 1B): i) cells were treated with vehicle or with 1 µm mapacalcine but they were not submitted to OGD. ii) cells were treated with vehicle or 1 µM mapacalcine during the 2 hours of OGD. iii) cells were treated with vehicle or 1 µM mapacalcine during 2 hours after the 2 hours of OGD.

Intracellular calcium concentration [Ca^2+^]_i_ was determined by monitoring the fluorescence intensity of a calcium indicator Fluo-2/AM under an inverted fluorescent microscope [25]. In brief, cortical neurons were seeded at a density of 1,000,000 cells/35-mm fluorodish. Cells were loaded 15–30 minutes with fluorescent probe Fura2-AM (10 µM) (Molecular probe, Invitrogen, Saint-Aubin, France) at 37°C in an atmosphere of 95% air/5% CO_2_ as previously described in [24]. Following 30 minutes for dye cleavage, the medium was replaced by cell calcium medium (CCM) containing (in mM) 116 NaCl, 5.6 KCl, 1.2 MgCl_2_, 2 CaCl_2_, 20 HEPES, 5 NaHCO_3_, 1 NaH_2_PO_4_. Then, fluorodishes were mounted on an inverted fluorescent microscope (Zeiss, France). Cells were imaged using a plan fluor 20X/0.75 oil/water immersion fluorescent objective at room temperature. Fura-2 probe was excited with alternating wavelengths 340/380 nm and images were acquired at 2s intervals. Intracellular calcium levels were recorded using a fluorimeter-based ratiometric system. For each assay (five different per condition), the number of analyzed cells were ranged between 400 and 1000 depending on the experimental condition. The fluorescence ratio at 340/380 nm provides an index of intracellular Ca^2+^ concentration. [Ca^2+^]_i_ was represented by the relative fluorescence intensity, δF/F0  =  (F-F0)/F0, where F is the fluorescence intensity measured after drug application, and F0 is the baseline. 6 different cultures (n = 6) were tested.

### OGD-induced cell injury: Cell survival and Lactate DeHydrogenase (LDH) measurements

#### Counting of cortical neurons by Hoechst staining

Cortical cells were treated with vehicle or 1 µM mapacalcine during OGD and for two hours after OGD (post treatment OGD) (Figure 1C). After OGD challenge the neuronal damage was assessed by Hoechst staining, which allowed to analyze the cell survival. Cells were washed with phosphate buffered saline solution (PBS, Invitrogen, Saint-Aubin, France) and were post-fixed with 4% paraformaldehyde (PFA) at 4°C. Then, cells were washed three times with PBS, incubated with 2 µg/mL Hoechst (Sigma-Aldrich, Saint-Quentin Fallavier, France) for 10 minutes and then washed with PBS. Nuclei of living cells were observed by using a videomicroscope with Metafluor software. Cell counting was made automatically by Image J software from 9 areas of 0.2 mm of diameter randomly chosen by the software. Survival neurons were counted in each experimental condition. Results were expressed as the number of cells per mm^2^ with standard error of the mean (SEM), (n = 24 wells, 9 fields per well for each condition in four different cultures).

#### Lactate deshydrogenase/Aqua Cell Titer (LDH/ACT) test

Cortical neurons were grown on poly D-lysine coated 24 well dishes. The 2 hours of OGD protocol was performed by 24 hours treatment with control solution alone or in the presence of 1 µM of mapacalcine or 1 µM of nifedipine. At the end of the treatment, both cell viability and cytotoxicity were measured by using the Cell Titer 96 (r) Aqueous One Solution Cell Proliferation (ACT), Assay (Promega, Charbonnières-les-Bains, France) and LDH release assay (Cytotoxicity detection kit, Roche diagnosis, Meylan, France), respectively. Protocols used were those described by manufacturers (n = 4 cultures, 24 wells per experimental group). ACT assay is a colorimetric method, which is based on the use of the 3-(4,5-dimethylthiazol-2-yl)-5-(3-carboxymethoxyphenyl)-2-(4-sulfophenyl)-2*H*-tetrazolium inner salt (MTS), a marker of mitochondrial activity and an electron-coupling reagent (phenazine ethosulfate, PES). The MTS tetrazolium compound is bioreduced by cells into a colored formazan product that is soluble in tissue culture medium. This conversion is presumably accomplished by NADpH or NADH produced by dehydrogenase enzymes in metabolically active cells. The quantity of formazan product as measured by the absorbance at 490 nm is directly proportional to the number of living cells in culture. According to the manufacturer's recommendations, the assay was performed as follows: the totality of cell culture medium was removed and replaced by 500 µl of Neurobasal medium + Cell Titer 96 Aqueous One Solution. Cells were incubated for 4 hours at 37°C in the humidified 5% CO_2_ atmosphere incubator. The reaction was stopped with 2% SDS. Optical density was measured 4 hours later at 490 nm utilizing a microplate reader (Labsystem Multiscan RC, VWR International, Fontenay sous Bois, France). Background absorbance at 620 nm was subtracted. Results were expressed in Optical Density (OD×10^−3)^. To correlate the mitochondrial activity measured by OD in the wells to the cell viability, a calibration curve was performed giving the effect of cell number on absorbance at 490 nm. The correlation coefficient was 0.99, indicating a linear response between cell number and absorbance at 490 nm. Data are expressed as the percentage of cell viability, which is calculated by dividing the absorbance value of mapacalcine/nifedipine-treated samples by that of the untreated control within each group. Neuronal injury was quantitatively assessed by the measurement of LDH release, which provides a measure of cytoplasmic membrane integrity. 100 µl of cell culture medium were transferred from culture wells to 96-well plates and mixed with 100 µl reaction solution according to LDH assay kit. Quantification was done by measuring the Optical Density (OD) 30 min later at 492 nm on a microplate reader (Labsystem Multiscan RC, VWR International, Fontenay sous Bois, France). Background absorbance at 620 nm was subtracted. As recommended by the manufacturer, neurons exposed to a lysis solution (PBS containing 0.1% Triton X-100) were used as positive control and set as 100% LDH release. Data are expressed as the ratio of LDH efflux/cell viability (n = 4 cultures, 24 wells per experimental group). All experiments were monitored by one researcher blinded to the treatment status. Results corresponded to the mean of four independent experiments with triplicate determination. Statistical analyses of cell viability and LDH results were assessed using one factor ANOVA test following by post-hoc test (*P*<0.05).

## Results

### Effect of mapacalcine on calcium currents

**Figure 2:**
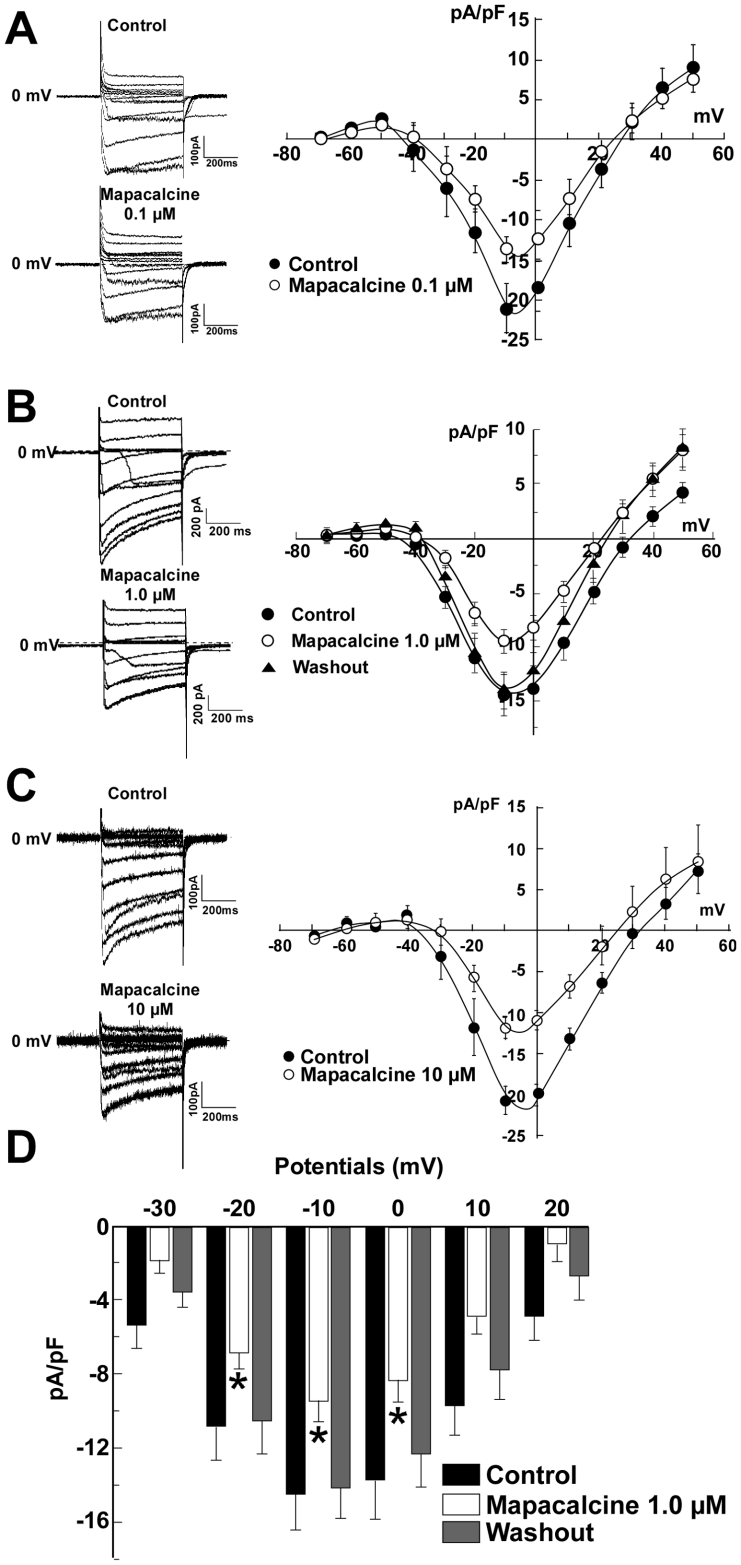
Dose-response effects of mapacalcine on calcium currents recorded in cortical neurons (**n = 10 for each dose**)**.** The holding potential was −80 mV. Calcium currents were recorded from −70 to +50 mV. (A) Calcium currents were recorded in control condition and in the presence of either 0.1 µM (A), or 1 µM (B) or 10 µM (C) of mapacalcine. In each condition, typical current traces and current densities (pA/pF) as a function of membrane potential (mV) are shown (D). Histograms of current densities measured at different potentials in control condition (black bars), in the presence of 1 µM of mapacalcine (white bars) and after washout (grey bars). Bars represent the SEM values. *, p<0.05.

**Figure 3:**
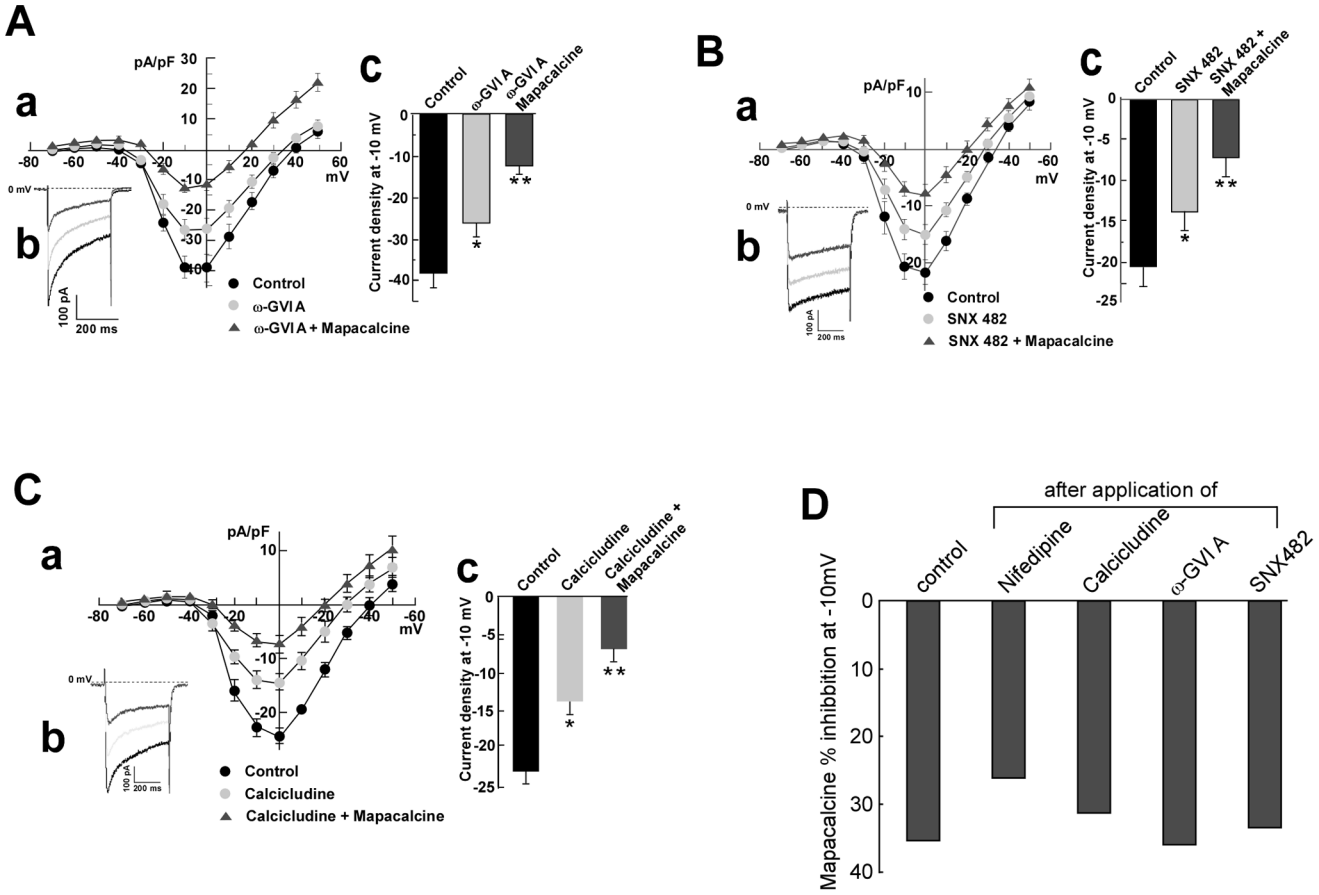
Effects of mapacalcine on electrophysiological recordings in cortical neurons in the presence of calcium channel blockers. (A) Effects of 1 µM of mapacalcine on remaining calcium current after application of 1 µM of ω-conotoxine GVI A, (a) I(pA/pF)  =  f(V) curves, (b) Typical current traces recorded at −10 mV, (c) Mean current traces at −10 mV. (B) Effects of 1 µM of mapacalcine on remaining calcium current after application of 250 nM SNX 482, (a) I(pA/pF)  =  f(V) curves, (b) Typical current traces recorded at −10 mV, (c) Mean current traces at −10 mV. (C) Effects of 1 µM of mapacalcine on remaining calcium current after application of 75 nM of calcicludine, (a) I(pA/pF)  =  f(V) curves, (b) Typical current traces recorded at −10 mV, (c) Mean current traces at −10 mV. (D) % of calcium current inhibition measured at −10 mV by mapacalcine when applied alone (control) or after previous application of the different toxine. Bars represent the SEM values. *, p<0.05, **, p<0.01. n = 10 for each experiment.

**Figure 4:**
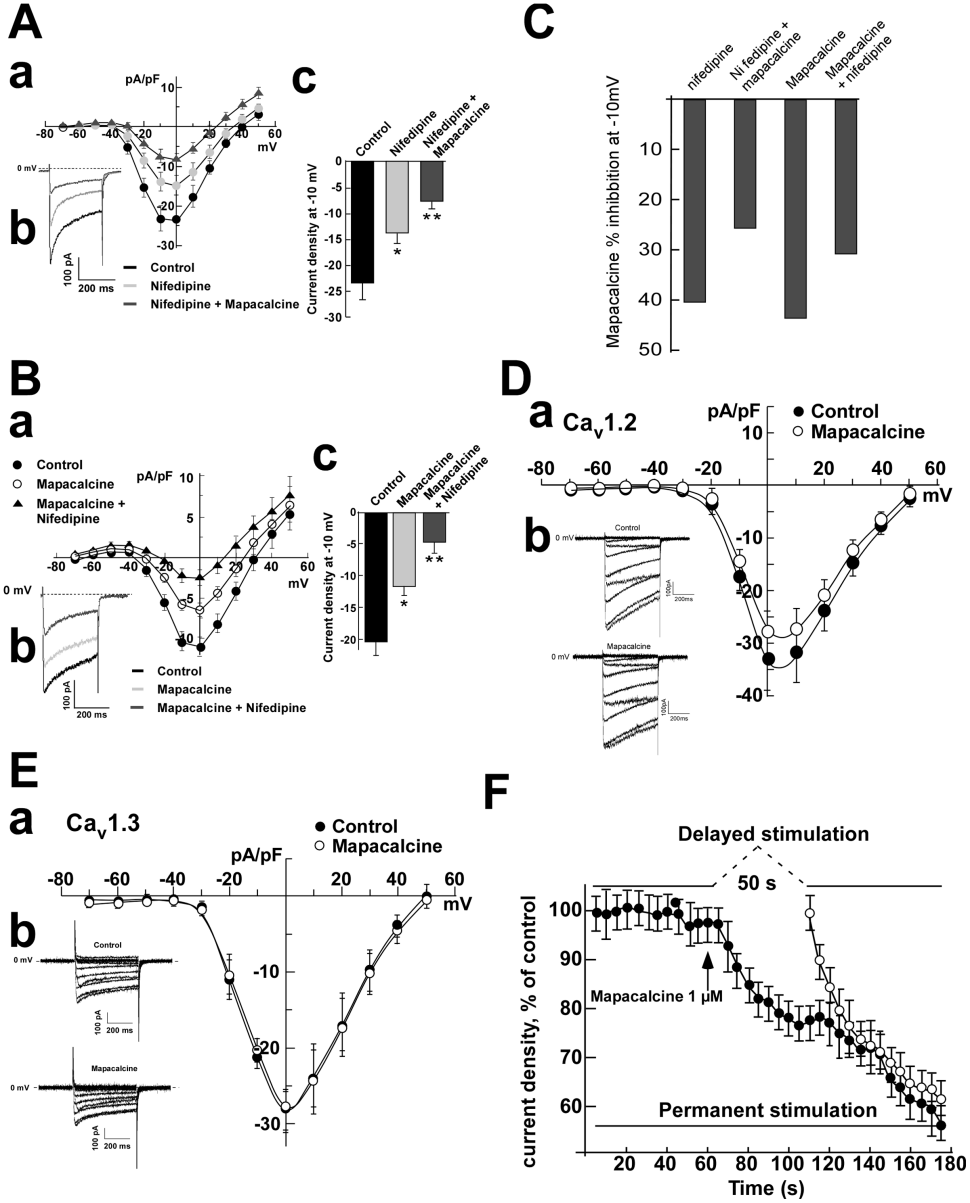
Effects of mapacalcine on electrophysiological recordings in cortical neurons or HEK-293 transfected cells. (A) Cortical neurons, effects of 1 µM of mapacalcine on remaining calcium current after application of 1 µM of nifedipine, (a) I(pA/pF)  =  f(mV) curves, (b) Typical current traces recorded at −10 mV, (c) Mean current traces at −10 mV. (B) Cortical neurons, effects of 1 µM of nifedipine on remaining calcium current after application of 1 µM of mapacalcine, (a) I(pA/pF)  =  f(V) curves, (b) Typical current traces recorded at −10 mV, (c) Mean current traces at −10 mV. (C) Comparaison of the % of inhibition of the calcium current measured at −10 mV by mapacalcine when it was applied after or before nifedipine. (D) HEK-293 transfected cells, effects of 1 µM of mapacalcine on Ca_V_ 1.2 calcium channels, (a) I(pA/pF)  =  f(V) curves, (b) Typical current traces. (E) HEK-293 transfected cells, effects of 1 µM of mapacalcine on Ca_V_ 1.3 calcium channels, (a) I(pA/pF)  =  f(V) curves, (b) Typical current traces. (F) Cortical neurons, effects of a delayed stimulation protocol on mapacalcine calcium channel inhibition. Each circle represent a stimulation cycle consisting in a 0.5 ms pulse from −80 to +10 mV, time between two pulses, 0.5 s. Bars represent the SEM values. *, p<0.05, **, p<0.01. n = 10 for each experiment.

Mapacalcine binds on calcium channels in peripheral tissues such as intestinal muscle [17], [18] and mapacalcine receptors were identified in brain [19]. Here, we investigated the effects of mapacalcine on calcium currents in mouse cortical neurons. Voltage-clamp recordings using the whole-cell configuration of the patch-clamp technique were first performed on cortical neurons in culture. Ca^2+^ current was evoked by step depolarization from −80 to +50 mV. Under these conditions, calcium currents started to activate at −40 mV and reached their maximum around −10 mV (Figure 2). Application of increasing concentrations of mapacalcine raised the current inhibition from 31% at 0.1 µM to 44% at 10 µM (n = 10 in each condition, Figure 2A–C). For example, 1 µM mapacalcine inhibited 35% of the calcium current generated at −10 mV (n = 10, Figure 2B,D). Then, we investigated the possibility that mapacalcine could compete with toxins known to be classical blockers of different voltage dependent calcium channel types. T-type calcium channels were excluded because their maximal peak amplitude are around −50 mV [26], [27], and in our experimental conditions there is no measurable currents at this potential (see Figure 2). It appeared that mapacalcine was unable to hinder or to increase the binding of drugs such as ω-conotoxin GVI A that is known to inhibit N-type calcium channel [28] (Figure 3A,D) or SNX 482 that inhibits the R-type calcium channel [29] (Figure 3B,D). Interestingly, in the presence of calcicludine that inhibits P/Q-type calcium channel [30] we observed a small decrease of the efficacy of mapacalcine (Figure 3C,D). This decreasing effect was more pronounced with nifedipine that inhibits L-type calcium channel [31] (Figure 4A,C). This effect was also present when drugs were applied in reverse. When mapacalcine was applied first, nifedipine inhibited in a lesser extend the channel activity confirming that both drugs shared a part of their binding sites (Figure 4A–C). Inhibitions of the total calcium current were calculated from mean values at −10 mV with mapacalcine alone or with mapacalcine after application of toxins or drugs (Figure 3D). Inhibition, expressed as a percentage of the total calcium current, due to mapacalcine application were 35% with mapacalcine alone, 35% for ω-conotoxin GVI A, 33% for SNX 482, 30% with calcicludine and 25% with nifedipine (Figure 3D). For nifedipine, the inhibition was 40.7% of the total current when it was applied directly on the stimulated channel and only reached 30% when mapacalcine was applied first (Figure 4A–C). These data clearly indicated that one of the mapacalcine targets is at least a neuronal subtype of L-type calcium channel. This is the first identification of a putative target for mapacalcine. The most important L-type subunit at the brain level is the subunit CaV1.3, but CaV1.2 subunit is also very important in brain or in heart [32], [33]. For these reasons we tested the effects of mapacalcine on both calcium channel subunits. Mapacalcine has no effect on either CaV1.2 activity, current values were of 33.02±5.83 pA/pF and 27.73±3.88 pA/pF for control and mapacalcine, respectively (Figure 4D), or CaV1.3 activity, current values were of 28.51±4.62 pA/pF and 28.22±5.17 pA/pF for control and mapacalcine, respectively (Figure 4E).

Additionally, we investigated whether the conformational state of the channel is important for the toxin binding. Two stimulation protocols, permanent and delayed, were used (Figure 4F). The permanent protocol indicated that when the membrane was depolarized by the potential jump to −10 mV, mapacalcine was rapidly efficient because a current inhibition appeared from the third stimulation cycle (Figure 4F). It appeared that in delayed stimulation protocol the starting level of inhibition was similar to the one obtained with permanent stimulation and, after few stimulation cycles of the delayed protocol, both curves (permanent and delayed) were superimposed. If the mapacalcine binding had been independent from the channel state we would have expected an inhibition value greater after fifty seconds of incubation with mapacalcine (*i.e* after the first restimulation cycle) than the value measured in permanent condition, but both were very close (Figure 4F).

These results indicated that mapacalcine is more efficient when the membrane is depolarized corresponding to an open state of calcium channels. The target channel of mapacalcine has to be in an open state for toxin binding.

### Effect of mapacalcine on intracytoplasmic calcium concentrations

**Figure 5:**
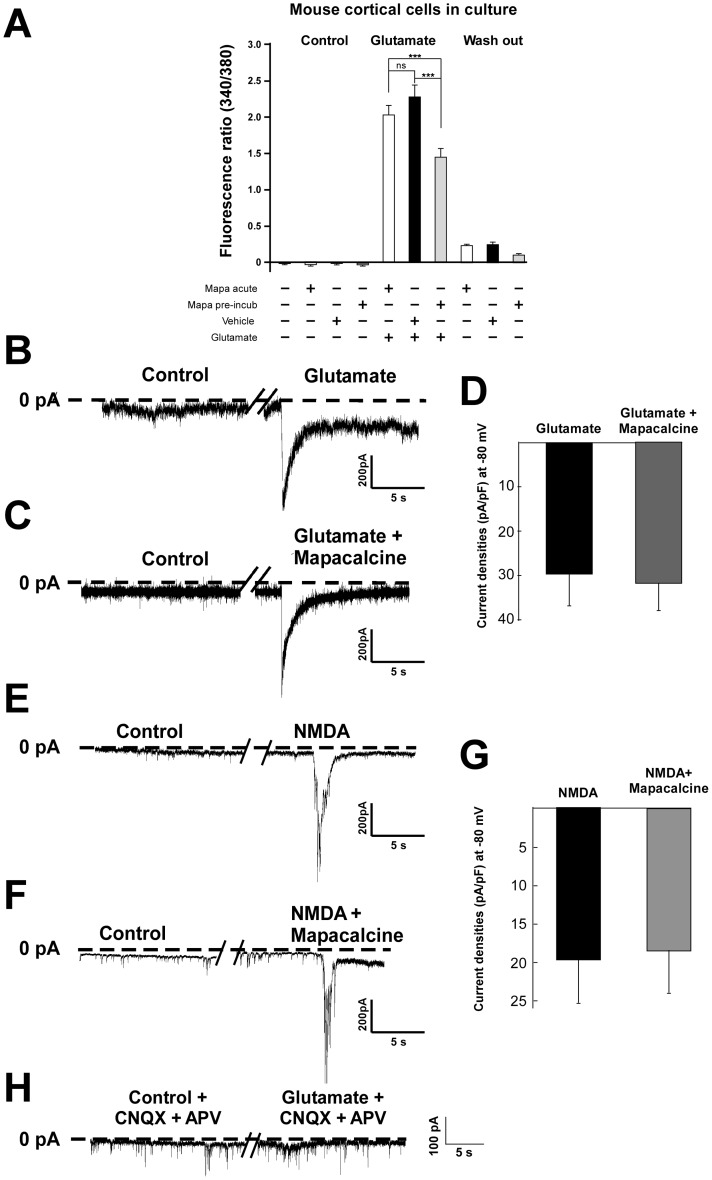
Calcium and electrophysiological measures after glutamate stimulation in cortical neurons. (A) The increase of intracellular calcium was triggered by application of 100 µM of glutamate. The different conditions are summarized under the histogram. *Mapa acute*, application of 1 µM of mapacalcine immediately before the measure. *Mapa pre-incub*, application of 1 µM of mapacalcine for 45 min before the measure. *Washout*, cells were perfused without glutamate. (B–H) Electrophysiological recording of glutamate currents (n = 6 per condition). (B) Typical current trace in control condition and after 100 µM of glutamate application. (C) Typical current trace in control condition and after 100 µM of glutamate application in the presence of 1 µM of mapacalcine. (D) Corresponding histogram of the current density values measured at the glutamate peak. (E) Typical current trace in control condition and after 100 µM of NMDA application. (F) Typical current trace in control condition and after 100 µM of NMDA application in the presence of 1 µM of mapacalcine. (G) Corresponding histogram of the current density values measured at the NMDA peak. (H) Typical current trace in control condition and after 100 µM of glutamate application in the presence of 10 µM of APV and 50 µM of CNQX inhibitors NMDA and AMPA/Kainate receptors, respectively (n = 10). Bars represent the SEM values. ***, p<0.001.

Since mapacalcine blocked a calcium current, we investigated its effect on the variation of the internal calcium concentration in control condition and following glutamate stimulation. In control condition, mapacalcine had no effect either after an acute application or 45 min pre-incubation (Figure 5A). After glutamate application, 1 µM mapacalcine had a slight reducing effect when applied in acute (*i.e*. measurements obtained few minutes after mapacalcine application). Normalized fluorescence ratio was 1.99±0.13 compared to 2.26±0.17 in the control (vehicle) condition (Figure 5A). This inhibitory effect was largely higher when mapacalcine was previously incubated for 45 min before the beginning of measurements. Normalized fluorescence ratio decreased to 1.42±0.12 compared to 2.26±0.17 in the vehicle condition (Figure 5A). These effects were reversible since after the washing out of glutamate, normalized fluorescence ratio reached values very close to basal values (Figure 5A). At this stage, we wondered whether these results were due to a blockade of glutamate receptors and more specifically of NMDA receptors. By using whole cell configuration of the patch clamp technique, we showed that mapacalcine had no effects on glutamate current or NMDA current (Figure 5B–G). At the day of culture we showed that the current initiated by glutamate application is inhibited by a mixture of APV (NMDA inhibitor) and CNQX (AMPA inhibitor) demonstrating the specificity of the glutamate-induced current (Figure 5H).

### Protective effects of mapacalcine against hypoxia

Glutamate release and intracellular calcium increase are two major consequences of hypoxia injuries [34], [35]. Then, we studied the effect of mapacalcine on OGD, an *in vitro* model of ischemia [23], [36], [37], [38].

**Figure 6:**
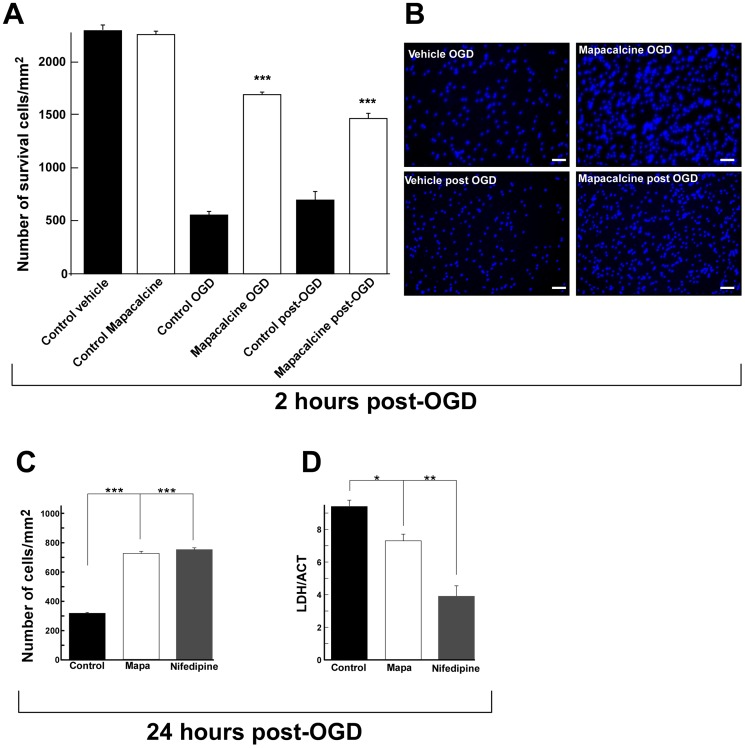
OGD on cortical neurons: cell survival. (A, B) Cell survival was determined after two hours OGD followed by two hours post treatment. (A) Histogram showing cell survival in the absence (control OGD) or the presence of 1 µM of mapacalcine (mapacalcine OGD). The number of cell survival was determinated after two hour OGD followed by two hour treatment with either vehicle (control post OGD) or 1 µM of mapacalcine (mapacalcine post OGD). (B) Typical picture of each condition obtained after Hoescht labeling of nuclei (scale bar  = 50 µM). (C–D) Cell survival was analyzed after two hours OGD followed by twenty four hours post treatment. (C) Histogram showing cell survival determined by counting, in control condition or in the presence of 1 µM of mapacalcine or 1 µM of nifedipine. (D) Histogram showing cell survival determined by LactateDesHydrogenase/AquaCellTiter ratio evaluations (LDH/ACT), in control condition and in the presence of 1 µM of mapacalcine or 1 µM of nifedipine. Values are the mean ± SEM (bars). *, p<0.05, **, p<0.01, *** p<0.001.

OGD protocol is considered as the best reliable *in vitro* model of ischemia [37], [38]. OGD consists in a glucose and oxygen deprivation, 1.2% instead of 5% in normal conditions. Mouse cortical neurons were incubated in different conditions, i) in the presence or ii) in the absence of 1 µM mapacalcine during the 2 hours of OGD and, iii) in the presence or iv) in the absence of 1 µM mapacalcine for 2 hours after OGD (protocols are schematized in Figure 1). At the end of each experimental condition, the number of Hoescht stained cells which survived was counted in 9 areas of the Petri dish. It clearly appeared that mapacalcine largely increased the cell survival (530.3±28.6 versus 1684.3±29.6 and 605.3±72.0 versus 1474.2±43.7 surviving cells/mm^2^ for mapacalcine application during and after OGD, respectively) (Figure 6A,B). We wondered whether this positive effect was really an action on the cell survival or simply a delayed effect. For this purpose, we measured remaining mapacalcine effects 24 hours post-OGD and as positive control we used a 1 µM nifedipine treatment. At this stage, it appeared clearly that the number of surviving cells is largely increased by both mapacalcine and nifedipine, values were of 319.84±5.9, 730.86±14.32 and 741.41±11.05 surviving cells per mm^2^ for control, mapacalcine and nifedipine condition, respectively (Figure 6C). These observations were confirmed by the measurement of LDH release which is a marker of cell suffering, taking in account both necrotic and apoptotic neuronal cell death. OGD induced an increase in LDH release. Addition of either mapacalcine or nifedipine reduced the LDH release/cell survival ratio 24 hours following OGD (Figure 6D).

These data demonstrated that mapacalcine displayed a true protective effect on cortical neuron survival that had undergone an OGD protocol.

**Figure 7:**
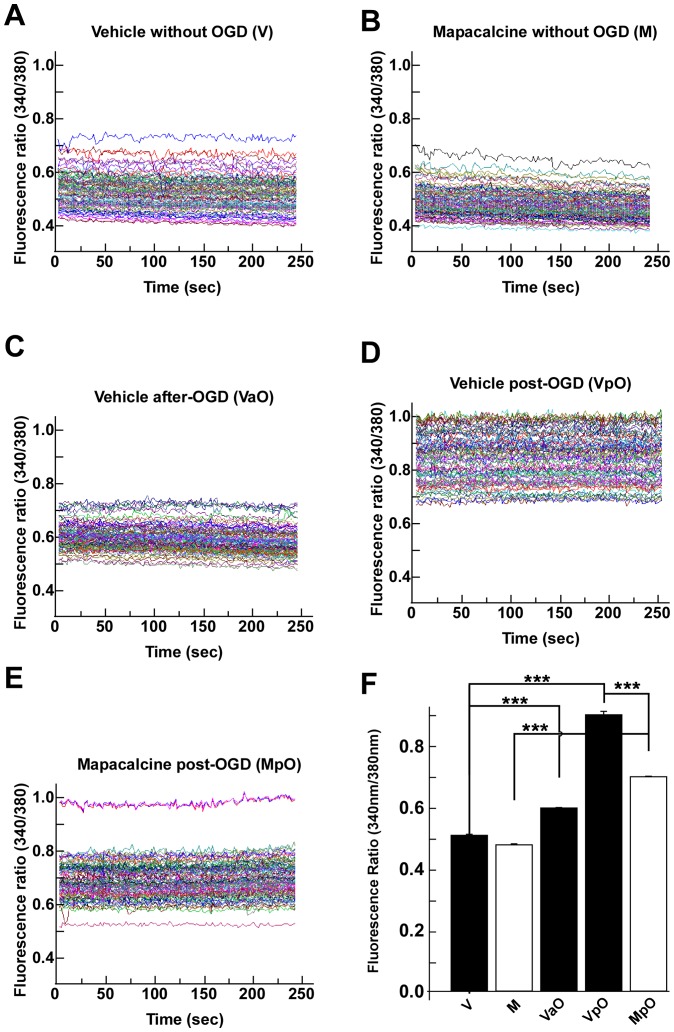
OGD on cortical neurons: calcium measures. Typical fluorescence ratio (340nm/380nm) obtained in different conditions. (A) Vehicle without OGD (V), (B) 1 µM mapacalcine without OGD (M), (C) Vehicle immediately after OGD (VaO), (D) Vehicle post-OGD (VpO), (E) Mapacalcine applied for two hours after OGD (MpO), (F) Corresponding histograms. Values are the mean ± SEM (bars). ***, p<0.001.

These protective effects were also evidenced by the calcium measurement technique. In the absence of OGD, the internal calcium concentrations were very similar in the presence or the absence of 1 µM mapacalcine (0.51±0.01 and 0.48±0.01, respectively) suggesting no toxic effect of mapacalcine (Figure 7A,B,F). But when the OGD protocol was applied, the calcium concentration in the vehicle treated cells was largely increased two hours after the end of OGD when compared to the measures performed immediately after OGD (fluorescence ratio of 0.91±0.02 versus 0.61±0.01, respectively) (Fig 7C,D,F). After two hours of treatment post-OGD mapacalcine reduced the calcium increase (fluorescence ratio of 0.70±0.01 instead of 0.91±0.02) (Figure 7E,F).

Taken together these results clearly demonstrate a protective effect of mapacalcine against ischemic conditions in neuronal cells.

## Discussion

Despite decades of research, stroke remains very difficult to be treated and attempts of developing effective treatments were unsuccessful, mainly because of the paucity of putatively efficient molecules tested. Actually, the main treatment against stroke is thrombolysis with recombinant plasminogen. This treatment has to be applied within 3 to 5 hours after stroke onset and remains restricted to a small number of patients.

Many attempts are worldwide conducted to identify new drugs and whenever possible, natural molecules such as compounds of the traditional Chinese Medicine [21], [39] or toxins [17], [40]. Animal toxins represent a large tank of molecules with potential therapeutic applications. For example toxins from snake or marine snail or spider, are widely used for studies on inflammation or pain [41], [42], [43]. Mapacalcine which has been purified from a natural sponge also displays very interesting therapeutical features. On rat hepatocytes, mapacalcine was already described to hinder calcium influx triggered by hypoxia [17] and in this study we demonstrated that mapacalcine could also be protective in neurons submitted to an OGD. We have first identified a target for mapacalcine, most probably a neuronal subtype of L-type calcium channel. This conclusion is based on the fact that in the presence of nifedipine, a specific blocker of L-type calcium channel, the percentage of calcium current blocked by the mapacalcine was lower than that measured when mapacalcine was applied alone (25% versus 35% respectively) and conversely when mapacalcine was added first nifedepine inhibited only 30% of channel activity instead of 40% when it was applied first. The decreased inhibitory effect is less pronounced with calcicludine which is an inhibitor of high threshold calcium channels which include L-type but also P/Q type calcium channels [30]. Mapacalcine did not interfere with specific inhibitors of N-type calcium channels, w-conotoxin GVI A, or R-type calcium channels, SNX 482.

We also demonstrated that the target channel of mapacalcine have to be in an open state to efficiently bind the toxin. Such a difference was already described for dihydropyridine compounds on L-type calcium channels [44].

These results are specific for the Central Nervous System because on peripheral tissues such as intestine, mapacalcine was unable to inhibit L-type calcium channels. On kidney, specific effectors of L-type calcium channels were unable to compete with mapacalcine [16], [18]. The question is to know what type of mapacalcine sensitive calcium channel may be involved in cell protection after OGD in peripheral tissues. At least two hypotheses could explain these observations i) the mapacalcine sensitive L-subtype calcium channel would be absent on peripheral tissues suggesting that mapacalcine would protect cells like hepatocytes from OGD by blocking another calcium entry pathway. ii) the mapacalcine sensitive L-subtype calcium channel would represent a minor part of the L-types calcium channels present on peripheral tissues being thus undetectable with respect to the sensitivity of the techniques used in [17]–[19]. However, mapacalcine blocking of a L-type calcium channels subtype can hardly account by itself for the protective effects observed here. Further experiments will have to be performed to identify all the biological targets involved in this protection.

Mapacalcine receptors have already been described in brain [19]. Data presented in this previous work showed that mapacalcine binds to specific receptors and that oxodipin and elgodipin, two L-type calcium channel blockers are unable to compete with mapacalcine. The discrepancy between these data and those reported here may be explained by the fact that experimental conditions used for binding experiments are very different from those used in this work for electrophysiological experiments. Polarization level of membranes used for binding was not controlled and had many chances to be out of the range at which the L-type calcium channel subtype highlighted here is open and accessible to mapacalcine. Data previously published and data reported in this work taken together would suggest that mapacalcine recognizes different receptors on nervous tissue, among which the particular subtype of L-type calcium channel described here. This L-type calcium channel subtype could represent only a small percentage of the total mapacalcine binding and thus be undetectable in binding experiments. This hypothesis is argued by the fact that mapacalcine is without effect on both Ca_V_1.2 and Ca_V_1.3 that are highly expressed in brain [32], [33]. Moreover, mapacalcine has also been shown to protect hepatocytes against hypoxia [17], consequently we wondered in this work whether mapacalcine could have a protecting effect on neurons against ischemia.

During stroke process, glutamate is released and induces an increase of intracellular calcium concentration [4]. The calcium entry is mainly due to voltage dependent calcium channel activation. The glutamate-triggered calcium influx was significantly reduced by mapacalcine, indicating that mapacalcine could protect cells against an ischemic insult. Nevertheless, our data rule out the possibility that the protective action of mapacalcine could involve glutamate receptors such as AMPA or NMDA receptor-channels. In cortical neurons in culture, the ischemic insult was mimicked by the OGD protocol. As expected, mapacalcine largely increased the survival of the OGD submitted cells when applied either during OGD or for two hours after OGD. Additionally, a post-treatment for two hours after OGD with mapacalcine significantly reduced the calcium influx, confirming the interesting neuroprotective role of mapacalcine.

In conclusion, in this study we identified for the first time a subtype of L-type calcium channel as one target for mapacalcine. We also pointed out that the channel has to be in its opened state for mapacalcine binding. We demonstrated its protective role in OGD experiments, technique that mimics stroke in cell culture. However, many other studies will be necessary to clarify the protective role of mapacalcine in neuroprotection. Mainly, the efficacy window and the efficient dose of mapacalcine in in vivo models of stroke have to be defined. Nevertheless, mapacalcine remains a very promising molecule for stroke treatment.
