# Correction: Mapacalcine Protects Mouse Neurons against Hypoxia by Blocking Cell Calcium Overload

**DOI:** 10.1371/annotation/d84d3131-a400-4ca6-9c5c-9ab0be4a6dd5

**Published:** 2013-09-19

**Authors:** Hamid Moha ou Maati, Catherine Widmann, Djamila Sedjelmaci Bernard Gallois, Catherine Heurteaux, Marc Borsotto, Michel Hugues

Due to issues with the typesetting process, there were errors in the article.

The names of the third author, Djamila Sedjelmaci, and the fourth author, Bernard Gallois, were incorrectly joined in the byline. The correct citation is: Moha ou Maati H, Widmann C, Sedjelmaci D, Gallois B, Heurteaux C, et al. (2013) Mapacalcine Protects Mouse Neurons against Hypoxia by Blocking Cell Calcium Overload. PLoS ONE 8(7): e66194. doi:10.1371/journal.pone.0066194

In addition, the affiliations of the third and fourth authors were incorrectly omitted from the byline. Djamila Sedjelmaci is affiliated with 1, Institut de Pharmacologie Moléculaire et Cellulaire, Centre National de la Recherche Scientifique (UMR7275), Université de Nice Sophia Antipolis, Valbonne, France. Bernard Gallois is affiliated with 2, Chimie Biologie des Membranes et des Nanoobjets, Centre National de la Recherche Scientifique (UMR5248), Pessac, France. 

